# Supplementary material for: A blood-based miRNA signature with prognostic value for overall survival in advanced stage non-small cell lung cancer treated with immunotherapy
Source: NPJ Precis Oncol. 2022 Mar 31;6:19. doi: 10.1038/s41698-022-00262-y (PMC8971493; doi:10.1038/s41698-022-00262-y)
Supplement: Supplementary file 3 — REPORTING SUMMARY [file 41698_2022_262_MOESM3_ESM.pdf]

nature portfolio

Corresponding author(s): Dr Bruno Steinkraus

Last updated by author(s): Jan 28, 2022

Reporting Summary

Nature Portfolio wishes to improve the reproducibility of the work that we publish. This form provides structure for consistency and transparency in reporting. For further information on Nature Portfolio policies, see our Editorial Policies and the Editorial Policy Checklist.

Statistics

For all statistical analyses, confirm that the following items are present in the figure legend, table legend, main text, or Methods section.

n/a	Confirmed	
☐	☒	The exact sample size (*n*) for each experimental group/condition, given as a discrete number and unit of measurement
☐	☒	A statement on whether measurements were taken from distinct samples or whether the same sample was measured repeatedly
☐	☒	The statistical test(s) used AND whether they are one- or two-sided<br>*Only common tests should be described solely by name; describe more complex techniques in the Methods section.*
☐	☒	A description of all covariates tested
☐	☒	A description of any assumptions or corrections, such as tests of normality and adjustment for multiple comparisons
☐	☒	A full description of the statistical parameters including central tendency (e.g. means) or other basic estimates (e.g. regression coefficient) AND variation (e.g. standard deviation) or associated estimates of uncertainty (e.g. confidence intervals)
☐	☒	For null hypothesis testing, the test statistic (e.g. *F*, *t*, *r*) with confidence intervals, effect sizes, degrees of freedom and *P* value noted<br>*Give P values as exact values whenever suitable.*
☒	☐	For Bayesian analysis, information on the choice of priors and Markov chain Monte Carlo settings
☒	☐	For hierarchical and complex designs, identification of the appropriate level for tests and full reporting of outcomes
☐	☒	Estimates of effect sizes (e.g. Cohen's *d*, Pearson's *r*), indicating how they were calculated

Our web collection on statistics for biologists contains articles on many of the points above.

Software and code

Policy information about availability of computer code

Data collection	Clinical data and laboratory results were collected by a systematic review of patient records, as described (Wessels et al., 2020).
Data analysis	Data analysis was performed in Python (3.8.8) with the packages scikit-learn (version 0.24.2), scikit-survival (version 0.15.1), and Lifelines (version 0.26.0), and RStudio (Version 1.3.1073) with the package timeROC (version 0.4).

For manuscripts utilizing custom algorithms or software that are central to the research but not yet described in published literature, software must be made available to editors and reviewers. We strongly encourage code deposition in a community repository (e.g. GitHub). See the Nature Portfolio guidelines for submitting code & software for further information.

Data

Policy information about availability of data

All manuscripts must include a data availability statement. This statement should provide the following information, where applicable:

- Accession codes, unique identifiers, or web links for publicly available datasets
- A description of any restrictions on data availability
- For clinical datasets or third party data, please ensure that the statement adheres to our policy

Anonymized small RNA sequencing data are available with controlled access approval through the European Nucleotide Archive under accession number PRJEB50502. All other data supporting the findings of this study are available from the corresponding author on reasonable request.

Field-specific reporting

Please select the one below that is the best fit for your research. If you are not sure, read the appropriate sections before making your selection.

☒ Life sciences ☐ Behavioural & social sciences ☐ Ecological, evolutionary & environmental sciences

For a reference copy of the document with all sections, see nature.com/documents/nr-reporting-summary-flat.pdf

Life sciences study design

All studies must disclose on these points even when the disclosure is negative.

Sample size	All analysis was performed on prospectively collected, biobanked samples that had been split into independent cohorts. All samples were analysed in order to maximise power.
Data exclusions	A single patient sample was excluded from the qPCR validation experiment for reason of insufficient RNA. A single patient was excluded from the miRNA expression to blood count correlation analysis for reason of being an outlier (5.2 standard deviations above the mean).
Replication	Replicate patient samples were not available. Experimental findings were confirmed through the analysis of an independent validation patient cohort.
Randomization	Patient samples were collected prospectively. Patients were randomised into training and validation cohorts in the clinic.
Blinding	Clinical data collection and analysis was performed blind to outcomes.

Reporting for specific materials, systems and methods

We require information from authors about some types of materials, experimental systems and methods used in many studies. Here, indicate whether each material, system or method listed is relevant to your study. If you are not sure if a list item applies to your research, read the appropriate section before selecting a response.

Materials & experimental systems

n/a	Involved in the study	
☒	☐	Antibodies
☒	☐	Eukaryotic cell lines
☒	☐	Palaeontology and archaeology
☒	☐	Animals and other organisms
☐	☒	Human research participants
☐	☒	Clinical data
☒	☐	Dual use research of concern

Methods

n/a	Involved in the study	
☒	☐	ChIP-seq
☒	☐	Flow cytometry
☒	☐	MRI-based neuroimaging

Human research participants

Policy information about studies involving human research participants

Population characteristics	The 96 patients in the training cohort of stage IV NSCLC patients who received anti-PD-1 monotherapy comprised 77% (n = 74) pembrolizumab- and 23% (n = 22) nivolumab-treated; 58% adenocarcinomas (n = 56), 28% squamous cell carcinomas (n = 27), and 14% other NSCLCs (n = 13, including NSCLC, NOS and large-cell neuroendocrine lung carcinomas); 62.5% males; 91.7% former or current smokers (Table 1). Immunotherapy was administered in the first line to 49%, in the second line to 48%, and beyond the second-line to only 3% of the patients (Table 1). 71% had PD-L1 TPS ≥ 50%, 21% 1-49%, and 8% <1%, while ECOG performance status (PS) at the time of immunotherapy start was 0 in 37%, 1 in 58%, and 2 in 5% of cases. The clinical characteristics of the independent validation cohort are similar and shown in Table 1. The independent control cohort consisted of 139 stage IV NSCLC patients treated with combined chemoimmunotherapy but who otherwise display broadly similar clincopathological features (Table 1).
Recruitment	Clinical samples were collected prospectively as published (Wessels et al., 2020) and provided by the Lungenbiobank Heidelberg and Biobank Nord. The 15 Grosshansdorf patients were recruited prospectively.
Ethics oversight	This study was approved by the Heidelberg University (S-296/2016, S-089/2019) and Grosshansdorf Hospital ethics committee (AZ 12-238 and AZ 19-268).

Note that full information on the approval of the study protocol must also be provided in the manuscript.

Clinical data

Policy information about clinical studies

All manuscripts should comply with the ICMJE guidelines for publication of clinical research and a completed CONSORT checklist must be included with all submissions.

Clinical trial registration	DRKS00018784, DRKS00022300
Study protocol	The study protocols are available upon request.
Data collection	Clinical data and laboratory results were collected by a systematic review of patient records, as described (Wessels et al., 2020).
Outcomes	Overall Survival (OS) from the time of therapy commencement was used as the endpoint.
